# Lactated Ringers, albumin and mannitol as priming during cardiopulmonary bypass reduces pulmonary edema in rats compared with hydroxyethyl starch

**DOI:** 10.1186/s40635-024-00661-4

**Published:** 2024-09-07

**Authors:** Anne M. Beukers, Anoek L. I. van Leeuwen, Roselique Ibelings, Anita M. Tuip-de Boer, Carolien S. E. Bulte, Susanne Eberl, Charissa E. van den Brom

**Affiliations:** 1 Department of Anesthesiology, Amsterdam UMC, VU University, Amsterdam, The Netherlands; 2 Department of Cardiothoracic Surgery, Amsterdam UMC, University of Amsterdam, Amsterdam, The Netherlands; 3 Laboratory for Experimental Intensive Care and Anesthesiology (LEICA), Amsterdam UMC, University of Amsterdam, Amsterdam, The Netherlands; 4 Department of Intensive Care Medicine, Amsterdam UMC, University of Amsterdam, De Boelelaan 1117, 1081 HV Amsterdam, The Netherlands; 5 Department of Anesthesiology, Amsterdam UMC, University of Amsterdam, Amsterdam, The Netherlands

**Keywords:** Cardiopulmonary bypass, Edema, Glycocalyx, Microcirculation, Priming

## Abstract

**Background:**

Endothelial disorders with edema formation and microcirculatory perfusion disturbances are common in cardiac surgery with cardiopulmonary bypass (CPB) and contribute to disturbed tissue oxygenation resulting in organ dysfunction. Albumin is protective for the endothelium and could be a useful additive to CPB circuit priming. Therefore, this study aimed to compare organ edema and microcirculatory perfusion in rats on CPB primed with lactated Ringers, albumin and mannitol (LR/albumin/mannitol) compared to 6% hydroxyethyl starch (HES).

**Results:**

Male rats were subjected to 75 min of CPB primed with either LR/albumin/mannitol or with 6% HES. Renal and lung edema were determined by wet/dry weight ratio. Pulmonary wet/dry weight ratio was lower in rats on CPB primed with LR/albumin/mannitol compared to HES (4.77 [4.44–5.25] vs. 5.33 [5.06–6.33], *p* = 0.032), whereas renal wet/dry weight ratio did not differ between groups (4.57 [4.41–4.75] vs. 4.51 [4.47–4.73], *p* = 0.813). Cremaster microcirculatory perfusion was assessed before, during and after CPB with intravital microscopy. CPB immediately impaired microcirculatory perfusion compared to baseline (LR/albumin/mannitol: 2 [1–7] vs. 14 [12–16] vessels per recording, *p* = 0.008; HES: 4 [2–6] vs. 12 [10–13] vessels per recording, *p* = 0.037), which persisted after weaning from CPB without differences between groups (LR/albumin/mannitol: 5 [1–9] vs. HES: 1 [0–4], *p* = 0.926). In addition, rats on CPB primed with LR/albumin/mannitol required less fluids to reach sufficient flow rates (0.5 [0.0–5.0] mL vs. 9 [4.5–10.0], *p* < 0.001) and phenylephrine (20 [0–40] µg vs. 90 [40–200], *p* = 0.004). Circulating markers for inflammation (interleukin 6 and 10), adhesion (ICAM-1), glycocalyx shedding (syndecan-1) and renal injury (NGAL) were determined by ELISA or Luminex. Circulating interleukin-6 (16 [13–25] vs. 33 [24–51] ng/mL, *p* = 0.006), interleukin-10 (434 [295–782] vs. 2120 [1309–3408] pg/ml, *p* < 0.0001), syndecan-1 (5 [3–7] vs. 15 [11–16] ng/mL, *p* < 0.001) and NGAL (555 [375–1078] vs. 2200 [835–3671] ng/mL, *p* = 0.008) were lower in rats on CPB primed with LR/albumin/mannitol compared to HES.

**Conclusion:**

CPB priming with LR, albumin and mannitol resulted in less pulmonary edema, renal injury, inflammation and glycocalyx degradation compared to 6% HES. Furthermore, it enhanced hemodynamic stability compared with HES. Further research is needed to explore the specific role of albumin as a beneficial additive in CPB priming.

## Introduction

Endothelial disorders with consecutive edema formation and microcirculatory perfusion disturbances are well-known phenomena in cardiac surgery with cardiopulmonary bypass (CPB) and contribute to a disturbed tissue oxygenation resulting in organ dysfunction [1–3]. CPB is associated with endothelial activation, hyperpermeability and vascular leakage in rats [4, 5] and patients [6] as a consequence of, among others, systemic inflammation and hemodilution [7], both of which are still inevitable with the use of CPB [2, 8, 9]. This results in accumulation of interstitial fluid and edema, which can compromise microcirculatory perfusion [10] and transport of oxygen and nutrients from blood to tissue [1], with kidney and lungs as most vulnerable organs [2, 3]. Several strategies are discussed to reduce these complications. In rats, pharmacologically protecting the endothelium was promising in reducing vascular leakage and preserving microcirculatory perfusion [4]. Interestingly, the choice of CPB priming might also play an important role in reduction of vascular leakage and consequently tissue edema. Different prime fluid strategies have been used in order to reduce interstitial fluid accumulation. These strategies mainly focused on preserving colloid oncotic pressure (COP), an important determinant of interstitial fluid accumulation [11], with a crystalloid or colloid-based prime fluid strategy [12–16]. It has previously been shown that human albumin and hydroxyethyl starch (HES) prevented an increase in extravascular lung water index compared to crystalloids [12, 14]. Albumin could be beneficial in an optimal priming strategy due to its protective effect on the endothelial glycocalyx, its ability to preserve COP, and minimize interstitial fluid accumulation [17, 18]. Furthermore, albumin reduces the risk of myocardial injury during cardiac surgery [17, 19]. HES has been withdrawn from the European market in 2013 by the European Medicines Agency’s (EMA) due to safety concerns. In the US, the Food and Drug Administration (FDA) issued in the same year a warning about the increased risk of mortality and renal injury and in Australia, the Therapeutic Goods Administration (TGA) restricted its use since 2018. Nevertheless, it is still used in many countries, such as New Zealand, mainly due to its cost-effectiveness compared with albumin, which was also shown in a recent meta-analysis [20]. Thus, compared to HES, human albumin is more expensive, but appears to confer benefits in terms of endothelial protection. However, it is unknown whether the use of albumin in CPB priming is beneficial in the reduction of organ edema and preservation of microcirculatory perfusion. Therefore, we aimed to investigate the effect of lactated Ringer, albumin and mannitol priming on lung and renal edema formation and microcirculatory perfusion in rats on CPB compared to HES priming. We hypothesize that priming with lactated Ringer, albumin and mannitol is beneficial for organ edema and microcirculatory perfusion in rats on CPB. Despite its higher costs, albumin may offer greater advantages in the context of cardiac surgery, compared with HES.

## Materials and methods

### Animals

**Fig. 1:**
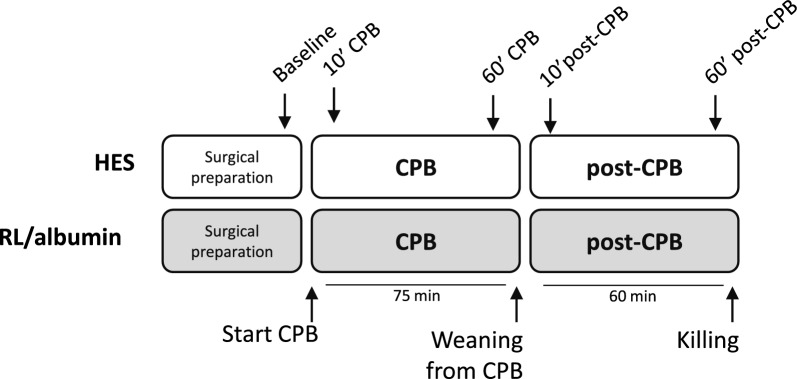
Experimental set-up. Rats were subjected to 75 min of CPB primed with LR/albumin/mannitol or HES. One hour after weaning from CPB, rats were killed to determine pulmonary and renal edema. Microcirculatory perfusion measurements were performed at baseline, 10 and 60 min after initiation of CPB and 10 and 60 min after weaning from CPB

All procedures were approved by the Institutional Animal Care and Use Committee of the VU University, the Netherlands (Animal welfare number: AVD1140020172144), and conducted following the EU Directive (2010/63EU) on the protection of vertebrate animals used for experimental and other scientific purposes and the Animal Research Reporting of In Vivo Experiments (ARRIVE) guidelines on animal research [21]. Male Wistar rats weighing 350–425 g (8–10 weeks of age, Charles River Laboratories, Brussels, Belgium) were housed in a temperature-controlled room (12/12 h light dark cycle, 20–23 °C, 40–60% humidity) with food and water ad libitum. Rats were not randomized between groups, the first rats (*n* = 9) were assigned to the CPB primed with HES, and the second (*n* = 9) to the CPB primed with a mixture of lactated Ringers, albumin and mannitol (LR/albumin/mannitol). Rats were subjected to 75 min of CPB. One hour following weaning from CPB, rats were killed by blood withdrawal under 5.0% isoflurane inhalation (Fig. 1). Kidneys and lungs were isolated and blood samples were collected and stored at − 80 °C for additional molecular analyses. Primary outcome was wet/dry weight ratio of lungs and kidneys. Secondary parameters included hemodynamic and blood gas parameters, cremaster microcirculatory perfusion, plasma levels of inflammatory markers: interleukin-6 (IL-6), interleukin-10 (IL-10), adhesion: intercellular adhesion molecule 1 (ICAM-1), glycocalyx degradation: syndecan-1, and renal injury: neutrophil gelatinase-associated lipocalin (NGAL), and fluid requirements. All outcome measures were measured and analyzed by an investigator who was blinded for intervention allocation.

### Priming

In the control group, the CPB circuit was primed with ± 10 mL 6% HES 130/0.4 (Voluven, Fresenius Kabi, Halden, Norway). In the intervention group, priming consisted of a mixture of lactated Ringer (LR; Baxter BV, Utrecht, the Netherlands), human albumin (Alburex 200 g/L, CSL Behring, King of Prussia, Pennsylvania, USA) and mannitol (15%, Baxter BV, Utrecht, the Netherlands) in a 85%, 10%, 5% ratio, respectively.

### Anesthesia and surgical preparation

All animals were anesthetized as previously reported [4, 5, 8] with 4.0% isoflurane (Ivax Farma, Haarlem, The Netherlands) in a plastic box filled with oxygen-enriched air. Following endotracheal intubation with a 14G catheter (Venflon Pro, Becton Dickinson, Helsingborg, Sweden), lungs were mechanically ventilated (UMV-03, UNO Roestvaststaal BV, Zevenaar, The Netherlands; PEEP 2–4 cm H_2_O, respiratory rate of 65–75 breaths/min, tidal volume ~ 10 ml/kg) and anesthesia was maintained with 1.5–2.0% isoflurane in oxygen-enriched air (40% O_2_/ 60% N_2_). Additionally, fentanyl (12 µg/kg, Janssen-Cilag, Tilburg, the Netherlands) was administered as additional analgesia and repeated approximately every 45 min during the experimental procedure. Respiratory rate was adjusted based on blood gas values to maintain pH and partial pressure of carbon dioxide within physiological limits. Depth of anesthesia was continuously monitored and adjusted if necessary based on heart rate and mean arterial pressure.

A 22G catheter (Venflon Pro, Becton Dickinson, Helsingborg, Sweden) was placed in the caudal (tail) or carotid artery for continuous measurements of arterial blood pressure and blood withdrawal for blood gas analysis and hematocrit measurements (ABL80, radiometer, Copenhagen, Denmark). Arterial blood pressure, electrocardiogram and heart rate were continuously recorded using PowerLab software (PowerLab 8/35, Chart 8.0; AD Instruments Pty, Ltd., Castle Hill, Australia).

The left cremaster muscle was isolated under warm saline superfusion, spread out on a heated platform (34 °C), and covered with gas impermeable plastic film (Saran wrap) for cremaster perfusion measurements as previously described [4, 5, 8, 22, 23].

Heparin (500 IU/kg, LEOPharma, Amsterdam, The Netherlands) was administered followed by cannulation of the right jugular vein with a modified multi-orifice 4.5 French catheter (Desilets-Hoffman, Cook, Bloomington, IN, USA) for venous outflow of the CPB circuit. The right femoral artery was cannulated with a 20G catheter (Arterial Cannula, Becton Dickinson, Helsingborg, Sweden) for arterial inflow of the CPB circuit. All catheter insertions were preceded by local application of 1% lidocaine.

Before initiation of the study protocol, an additional dose of heparin (500 IU/kg) was given in combination with rocuronium bromide (1.5 mg/kg, Organon, Oss, The Netherlands).

### Cardiopulmonary bypass

The protocol for CPB was performed as previously described [4, 5, 8]. In summary, the CPB circuit consists of an open venous reservoir, a roller pump (Pericor SF70, Verder, Haan, Germany), and an oxygenator-heat exchanger with a three-layer hollow fiber membrane for gas exchange (Ing. M. Humbs, Valley, Germany). A 1.0-mm-diameter arterial line (LectroCath, Vygon, Ecouen, France) was connected to the femoral inflow catheter. During CPB, CO_2_ and O_2_ pressures of the oxygenator membrane of the CPB circuit were adjusted based on blood gas values to maintain pH and partial pressure of carbon dioxide within physiological limits.

To maintain target CPB flow rates > 150 ml/kg/min, additional doses of HES in the control group or human albumin in the LR/albumin/mannitol group were administrated when necessary. If necessary, boluses of phenylephrine (10 µg) were administered to maintain mean arterial pressure above 50 mmHg to sustain organ perfusion pressure.

Weaning from CPB occurred after 75 min of extracorporeal circulation. The venous cannula was removed and the jugular vein was clamped. Fifteen min after weaning from CPB, protamine hydrochloride (2 mg/kg) was administered to neutralize heparin.

### Renal and pulmonary edema

Kidney and lung tissue was harvested at the end of the experiment under terminal anesthesia. Wet tissue was weighed and dried at 37 °C. After 1 week, dry tissue was weighed and wet/dry weight ratio was calculated as estimate for tissue water content.

### Cremaster microcirculatory perfusion

Microcirculatory perfusion measurements were performed using a 10× objective on an intravital microscope (AxiotechVario 100HD, Zeiss, Oberkochen, Germany) connected to a digital camera (scA640, Basler, Ahrensburg, Germany) with a final magnification of 640×, as described previously [4, 5, 8, 22, 23]. Briefly, three regions of the microvasculature (vessels up to 25 µm diameter) in the cremaster muscle with adequate perfusion quality were selected during baseline. These exact predefined regions were followed throughout the experiment: after surgical preparation of the cremaster muscle before onset of CPB (baseline), 10 min after initiation of CPB (10’ CPB), 60 min after initiation of CPB (60’ CPB), 10 min after weaning from CPB (10’ post-CPB) and 60 min after weaning from CPB (60’ post-CPB) (Fig. 1). For perfusion analyses, two vertical lines were drawn in each video screen. The total amount of capillaries per screen was obtained by averaging the counted capillaries crossing the two vertical lines. These small vessels were categorized as continuously perfused, intermittently perfused (blood flow was arrested at least once or flow was reversed), or non-perfused capillaries (vessels without erythrocytes or non-flowing erythrocytes). Finally, the proportion of continuously perfused vessels (PPV) was calculated by the ratio of the absolute number of continuously perfused vessels and the total number of vessels.

### Plasma analyses

Arterial blood was collected at baseline, 60 min after initiation of CPB (60’ CPB), and 60 min after weaning from CPB (60’ post-CPB). Plasma levels IL-6, IL-10, ICAM-1 were measured using a luminex platform (Biotechne). Plate-to-plate variation was accounted for using negative and positive controls. Values below the detection limit were imputed with the lower limit of quantification given by the calibration curve for the univariate comparisons. Measurements were judged to be unreliable when less than 25 beads were counted or no extrapolation outside of the reference standard concentrations and were therefore excluded for analysis. Plasma levels of syndecan-1 (MBS2703971, MyBioSource, San Diego, California, USA) and neutrophil gelatinase-associated lipocalin (NGAL; ab119602, Abcam, Cambridge, United Kingdom) as markers for glycocalyx degradation and renal injury, respectively, were measured with ELISA in accordance to the manufacturer.

### Statistical analysis

Sample size was calculated based on pilot experiments in which rats on CPB primed with lactated Ringer, albumin and mannitol had a lower lung wet/dry weight ratio (4.6 ± 0.4) compared to rats on CPB primed with HES (5.6 ± 0.8). To detect a difference in wet/dry weight ratio of 1.0, the two-sided significance level was set at 0.05. Using an alpha of 0.05 and a power of 0.90, a sample size of 9 rats per group was calculated.

All data are expressed as median [interquartile range] and analyzed using GraphPad Prism 9.0 (GraphPad Software, La Jolla, CA, USA). Non-parametric tests were used for all analyses because of small group sizes. Two-sided (multiple) Mann–Whitney *U* tests were used to evaluate differences between priming groups. Time-dependent (within group) differences were analyzed with a Friedman test with Dunn’s post hoc analyses. *P* values < 0.05 were considered as statistically significant.

## Results

### Hemodynamics and blood gas analysis

**Fig. 2:**
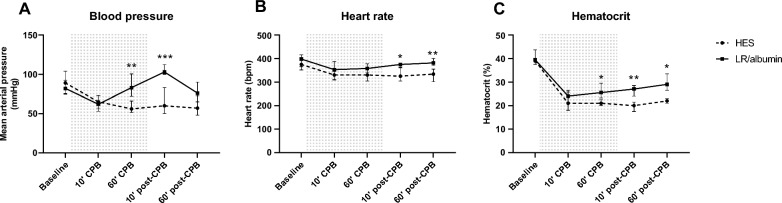
Hemodynamic parameters during CPB. Mean arterial pressure (**A**), heart rate (**B**) and hematocrit (**C**) in rats during and after CPB primed with LR/albumin/mannitol (continuous line, *n* = 9) or with HES (dashed line, *n* = 9). The grey background indicates the period during CPB. Data are presented as median with interquartile range, ** *p* < 0.01, *** *p* < 0.001, **** *p* < 0.0001 HES vs. LR/albumin/mannitol priming

Body weight of rats receiving LR/albumin/mannitol priming was lower compared to rats receiving HES priming (LR/albumin/mannitol: 379 [359–396] vs. HES: 401 [391–407] gram, *p* < 0.01). Initiation of CPB induced a drop in mean arterial pressure (LR/albumin/mannitol: 62 [57–73] vs. 82 [76–93] mmHg, *p* = 0.369; HES: 65 [53–73] vs. 89 [75–104] mmHg, *p* = 0.99; Fig. 2A), heart rate (LR/albumin/mannitol: 353 [309–388] vs. 398 [352–416] bpm, *p* = 0.369; HES: 330 [312–345] vs. 375 [366–398] bpm, *p* = 0.006; Fig. 2B) and hematocrit (LR/albumin/mannitol: 24 [22–26] vs. 40 [38–44] mmHg, *p* < 0.0001; HES: 21 [18–27] vs. 39 [38–40] %, *p* = 0.018; Fig. 2C), without differences between priming groups. Mean arterial pressure and heart rate restored in rats on CPB primed with LR/albumin/mannitol, but not in control rats (Fig. 2A and B). Unless differences in body weight, hematocrit levels were higher in rats on CPB primed with LR/albumin/mannitol compared to HES (29 [27–34] % vs. 22 [21–23] % vs. *p* = 0.011) (Fig. 2C).

**Fig. 3:**
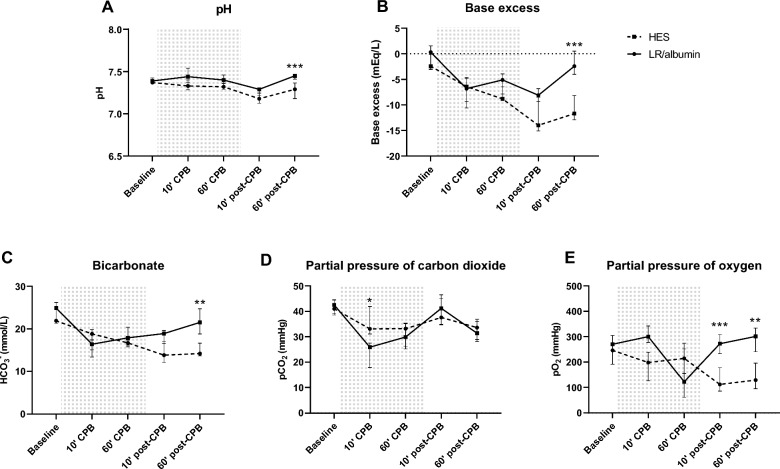
Blood gas analyses during CPB. pH (**A**), base excess (**B**), bicarbonate (HCO_3_^−^; **C**), partial pressure of carbon dioxide (pCO_2_; **D**) and partial pressure of oxygen (pO_2_; **E**) in rats during and after CPB primed with LR/albumin/mannitol (continuous line, *n* = 9) or HES (dashed line, *n* = 9). The grey background indicates the period during CPB. Data are presented as median with interquartile range, *** *p* < 0.001, **** *p* < 0.0001 HES vs. LR/albumin/mannitol priming

In addition, onset of CPB reduced bicarbonate levels, base excess and pCO_2_, but not pH which decreased following weaning from CPB in both priming groups (Fig. 3A–D). Interestingly, one hour following weaning from CPB, pH, base excess and bicarbonate levels restored in rats on CPB primed with LR/albumin/mannitol, but not in control rats on CPB primed with HES (Fig. 3A–C). Also differences were found in pO_2_ between priming groups, in which rats on CPB primed with LR/albumin/mannitol had higher pO_2_ levels after CPB compared to HES (Fig. 3E), without alterations in O_2_ saturation (98.0 [94.9–98.5] vs. 99.1 [96.5–99.6] %, *p* = 0.99).

### Microcirculatory perfusion

**Fig. 4:**
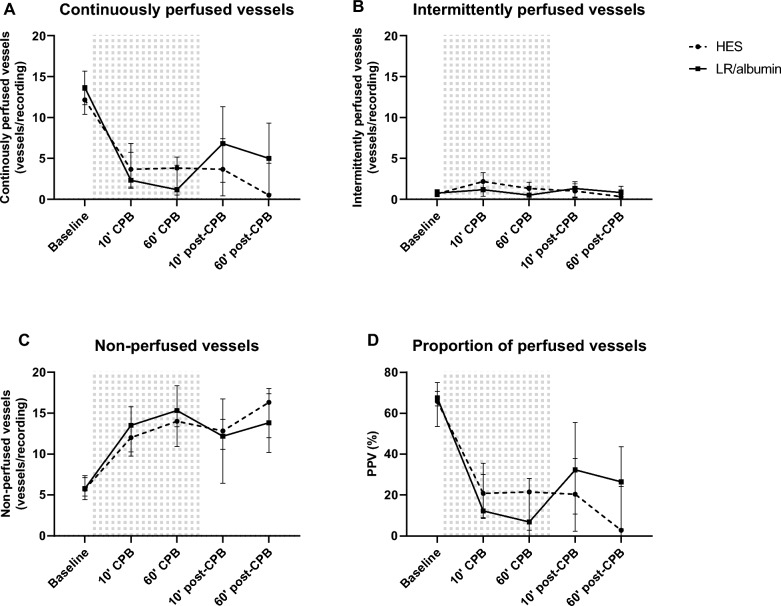
Microcirculatory perfusion. Continuously perfused vessels (**A**), intermittently perfused vessels (**B**), non-perfused vessels (**C**) and proportion of perfused vessels (PPV; **D**) in cremaster muscle in rats during and after CPB primed with LR/albumin/mannitol (continuous line, *n* = 9) or HES (dashed line, *n* = 9). The grey background indicates the period during CPB. Data are presented as median with interquartile range

Onset of CPB decreased the number of continuously perfused vessels (HES: 4 [2–6] vs. 12 [10–13] vessels per recording, *p* = 0.037; LR/albumin/mannitol: 2 [1–7] vs. 14 [12–16] vessels per recording, *p* = 0.008; Fig. 4A) and PPV (HES: 21 [9–30] vs. 66 [54–71] %, *p* = 0.018; LR/albumin/mannitol: 13 [9–36] vs. 68 [64–75] %, *p* = 0.037; Fig. 4D) compared to baseline. Also, onset of CPB increased the number of non-perfused vessels compared to baseline (HES: 12 [10–14] vs. 6 [5–7], *p* = 0.073; LR/albumin/mannitol: 14 [10–16] vs. 6 [4–7], *p* = 0.046; Fig. 4C). The number of continuously perfused vessels, proportion of perfused vessels and non-perfused vessels remained unaltered during and after CPB, without differences between priming groups (Fig. 4). No differences were found in intermittently perfused vessels over time and between priming groups (Fig. 4B).

### Lung and kidney edema formation and fluid requirements

**Fig. 5:**
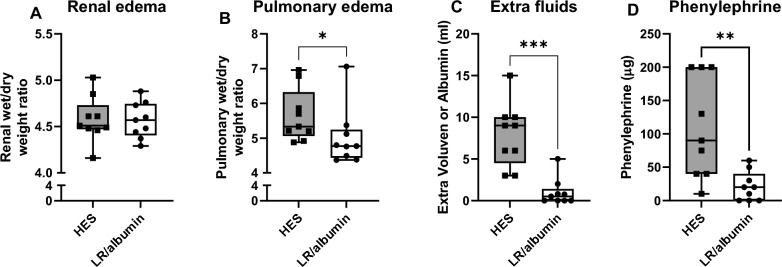
Renal and pulmonary edema formation and fluid requirements. Renal (**A**) and pulmonary (**B**) wet/dry weight ratio, extra fluids (**C**) and phenylephrine given during CPB (**D**) in rats following CPB primed with LR/albumin/mannitol (LR/a/m; white box) or HES (grey box). Rats on CPB primed with LR/albumin/mannitol received albumin and primed with HES received HES as additional fluids. Data are presented as median, interquartile and full range, every dot represents an individual rat, * *p* < 0.05, **, *p* < 0.01, *** *p* < 0.001 HES vs. LR/albumin/mannitol priming

Renal wet/dry weight ratio did not differ between priming groups (LR/albumin/mannitol: 4.57 [4.41–4.75] vs. HES: 4.51 [4.47–4.73], *p* = 0.813; Fig. 5A). Interestingly, rats on CPB primed with LR/albumin/mannitol had lower pulmonary wet/dry weight ratios compared to control rats on CPB primed with HES (4.77 [4.44–5.25] vs. 5.33 [5.06–6.33], *p* = 0.032; Fig. 5B). In addition, rats on CPB primed with LR/albumin/mannitol required less extra fluids (0.5 [0.0–1.4] vs. 9 [4.5–10.0] mL, *p* < 0.001, Fig. 5C) and less phenylephrine (20 [0–40] vs. 90 [40–200], *p* = 0.004, Fig. 5D) to maintain adequate CPB pump flow and a mean arterial pressure above 50 mmHg compared to control rats on CPB primed with HES. No differences were found in CPB pump flow (LR/albumin/mannitol: 69 [64–70] vs. HES: 62 [59–69] mL/min, *p* = 0.213) or administration of rocuronium (LR/albumin/mannitol: 0.9 [0.5–1.0] mL; HES: 0.8 [0.7–1.0] mL, *p* = 0.651).

### Circulating markers of inflammation, adhesion, glycocalyx shedding and renal injury

**Fig. 6:**
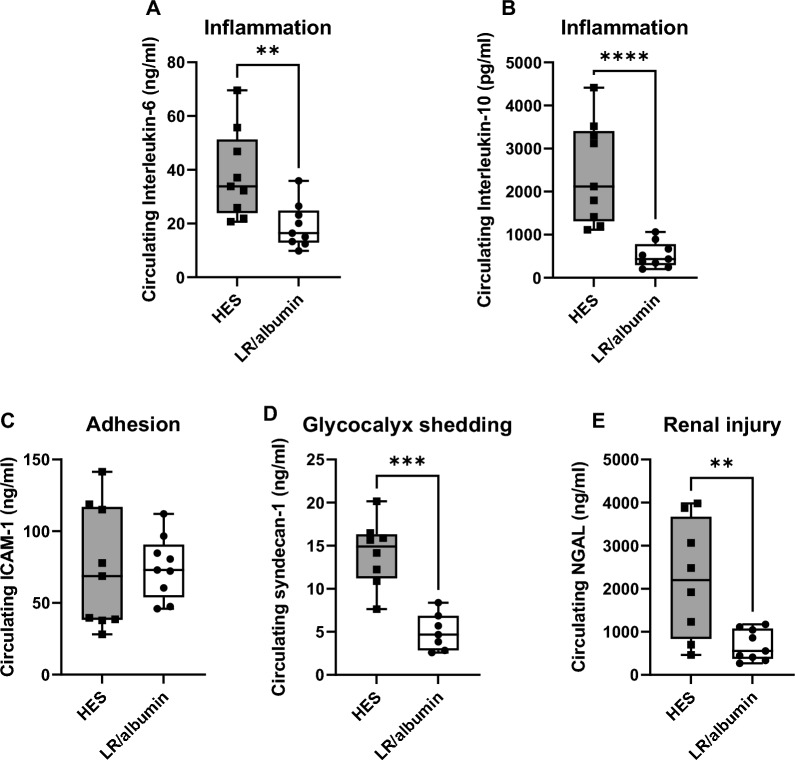
Circulating markers of inflammation, adhesion, glycocalyx shedding and renal injury. Plasma levels of interleukin-6 (IL-6; **A**), interleukin-10 (IL-10; **B**), intercellular adhesion molecule 1 (ICAM-1; **C**), syndecan-1 (**D**) and neutrophil gelatinase-associated lipocalin (NGAL; **D**) in rats during and after CPB primed with LR/albumin/mannitol (LR/a/m; white box) or HES (grey box). Data are presented as median, interquartile and full range, every dot represents an individual rat, ** *p* < 0.01, *** *p* < 0.001, **** *p* < 0.0001 HES vs. LR/albumin/mannitol priming

Circulating IL-6 (16 [13–25] vs. 34 [24–51] ng/mL, *p* = 0.006), IL-10 (434 [295–782] vs. 2120 [1309–3408] pg/ml, *p* < 0.0001), syndecan-1 (4.7 [2.8–6.9] vs. 14.9 [11.2–16.3] ng/mL, *p* < 0.001) and NGAL (555 [375–1078] vs. 2200 [835–3671] ng/mL, *p* = 0.008) were lower in rats on CPB primed with LR/albumin/mannitol compared to HES (Fig. 6A, B, D, E), but there were no differences in circulating levels of ICAM-1 (73 [54–91] vs. 69 [38–117] ng/mL, *p* = 0.666, Fig. 6D).

## Discussion

The main finding in this study is that CPB priming with LR/albumin/mannitol resulted in less pulmonary edema formation in rats on CPB compared with 6% HES priming, whereas priming strategy did not affect renal edema formation. In accordance, rats on CPB primed with LR/albumin/mannitol required less additional fluids, had higher mean arterial pressures and received less phenylephrine compared to rats on CPB primed with HES. Onset of CPB resulted in a rapid impairment of microcirculatory perfusion, which did not restore following weaning from CPB nor was affected by priming strategy. Interestingly, LR/albumin/mannitol priming resulted in less CPB-induced inflammation, glycocalyx degradation and renal injury.

Edema formation is common in cardiac surgery with CPB. Tissue edema increases the distance between capillaries which impairs oxygen delivery [24], and may contribute to organ failure. We found less pulmonary edema and fluid requirements in rats on CPB with LR/albumin/mannitol compared to HES priming. Previous literature showed beneficial effects of both colloids (HES and albumin) on extravascular lung water index compared with crystalloids [12, 14]. Despite a difference in oncotic force, both colloid fluids—albumin and HES–provide colloid oncotic force and thereby prevent edema formation [12, 14].

Explanation for the difference in edema formation could be the use of mannitol in the LR/albumin/mannitol priming strategy. Mannitol acts as a volume expander and is suggested to increase plasma osmolality [25]. This might explain why the LR/albumin/mannitol group required less fluids to maintain mean arterial pressure and CPB pump flow compared to the HES control group. Though, the beneficial effect of mannitol in CPB priming remains controversial. Endothelial cells might shrink due to fluid shifts from intracellular to extracellular, resulting in compromised wall integrity and increased permeability [25]. In a double-blind randomized controlled trial, the addition of mannitol to Ringers acetate did not influence osmolality [26], and its clinical impact on fluid balance in patients undergoing cardiac surgery remains debatable [27]. While it remains speculative, lungs might be more sensitive to inflammation and pulmonary edema, although no conclusive evidence supports this finding. In addition, during CPB, rats were ventilated—a potential secondary hit to the lungs—potentially contributing to increased inflammation and subsequent edema formation when compared to the kidneys. However, it is important to note that these statements remain speculative.

Nevertheless, albumin might have an additional beneficial effect by protecting the endothelial glycocalyx layer. In guinea pig hearts, albumin resulted after 4 h of cold ischemia in lower shedding of the glycocalyx [17]. In accordance, our study demonstrated that the use of LR/albumin/mannitol was paralleled by less glycocalyx shedding, as measured by lower circulating syndecan-1.

Importantly, NGAL, a marker released in the kidney in response to renal tubular injury, was higher in rats on CPB primed with HES. Renal NGAL increases rapidly after ischemia/reperfusion and has been described as a sensitive, specific and highly predictable marker of acute kidney injury after cardiac surgery [28]. Moreover, inflammation was lower in rats on CPB primed with LR/albumin/mannitol, which might explain the reduced pulmonary edema in this group. Interestingly, also the anti-inflammatory response was lower following LR/albumin/mannitol priming, whereas levels of ICAM-1, a marker of endothelial activation, were comparable. The reduced inflammatory response in the LR/albumin/mannitol priming group might be attributed to the prospective properties of albumin on the glycocalyx [17]. Taken together, these data suggest that priming with LR/albumin/mannitol compared to HES has protective effects on both lungs and kidneys in rats on CPB.

Microcirculatory perfusion disturbances are commonly seen in patients undergoing cardiac surgery with CPB [1, 6, 7, 10]. In the present study, onset of CPB resulted in a rapid fall of microcirculatory perfusion which persisted after weaning from CPB. These findings are in agreement with previous studies [4, 5, 8]. Colloids increase blood viscosity and may preserve microcirculatory perfusion through preservation of capillary pressure [29]. This could explain why changes in microcirculatory perfusion was not affected by prime fluid strategy. Yet, there are several factors known to contribute to CPB-induced microcirculatory perfusion disturbances [9, 29, 30]. First, hypotension and hemodilution [8] can both decrease capillary red blood cell flow and capillary vessel density, respectively [24], resulting in impaired oxygen delivery to tissues. Interestingly, rats on CPB primed with LR/albumin/mannitol had higher mean arterial pressures and hematocrit levels after weaning from CPB, but microcirculatory perfusion remained equally disturbed. No other surrogate markers for microcirculatory oxygen uptake were measured. Second, microcirculatory perfusion disturbances during CPB are associated with increased glycocalyx degradation [31]. The normal endothelial glycocalyx layer has the property to adjust its permeability to facilitate changes in red blood cell deformation and thereby counteract the increased heterogeneity of perfusion [32]. As a consequence of glycocalyx degradation, the adaptive ability of the microcirculation to changes in microvascular flow can be affected [33]. HES priming resulted in more glycocalyx degradation compared to LR/albumin/mannitol. Nevertheless, this did not affect microcirculatory perfusion. Although unexpected, differences in glycocalyx degradation without alterations in microcirculatory perfusion in cardiac surgery patients on CPB with different CPB circuit coatings have been reported earlier [31]. Although it is known that pharmacologic reduction of the inflammatory response preserves microcirculatory perfusion in rats during CPB, the inflammatory response was higher with HES without differences in microcirculatory perfusion [34]. In summary, CPB prime fluid strategies equally impair microcirculatory perfusion in rats independent of hemodynamic alterations, hemodilution and glycocalyx degradation.

Our study results suggest that the use of LR/albumin/mannitol in CPB priming might reduce the inflammatory response, preserve endothelial integrity and organ edema following CPB compared to HES priming. On the other hand, microcirculatory perfusion did not differ between priming strategies, although groups were not powered to for this outcome. Further research is needed to explore the specific role of albumin as part of CPB fluid priming as a viable strategy to maintain glycocalyx integrity and reduce organ injury. Though, future studies in adult cardiac surgery with CPB should elucidate whether albumin is a superior colloid to other colloids for CPB fluid priming regarding microcirculatory integrity and organ injury.

This study is performed in rats on extracorporeal circulation, which is a unique technique worldwide and contributes to our knowledge in the field of microcirculatory disorders in cardiac surgery. We acknowledge several limitations. Prime groups differed on more than one prime fluid component, not only the colloids. Mannitol in the albumin group could have confounded the affected outcomes. Mannitol was added from clinical perspective, consistent with our current clinical practice. Globally, its use is estimated at 30% in CPB priming [35]. Nevertheless, evidence regarding the mitigation of AKI [36, 37], the efficacy as free radical scavenger and its significance on fluids during cardiac surgery remains scarce (26). However, we acknowledge this a limitation to our approach. In addition, rats were not randomized between groups, this has potentially introduced bias.

In conclusion, priming of CPB with a mixture of LR/albumin/mannitol resulted in less pulmonary edema and renal injury compared to priming with HES priming and was paralleled by less inflammation and glycocalyx degradation. Furthermore, priming with LR/albumin/mannitol resulted in an improved hemodynamic stability compared with HES. In view of these and previous results, it certainly makes sense to think that albumin may improve oxygen delivery to tissues due to reduced organ edema. Therefore, albumin could be more cost effective in its use in CPB priming. Future research in cardiac surgery patients is needed to create an evidence-based prime fluid strategy to preserve microcirculatory oxygen delivery and thus organ function following CPB.

## Data Availability

The datasets used and/or analyzed during the current study are available from the corresponding author on reasonable request.

## Abbreviations

ARRIVE: Animal research reporting of in vivo experiments
CPB: Cardiopulmonary bypass
COP: Colloid oncotic pressure
ELISA: Enzyme-linked immuno sorbent assay
EMA: European Medicines Agency’s
FDA: Food and Drug Administration
HES: Hydroxyethyl starch
ICAM-1: Intercellular adhesion molecule 1
IL-6: Interleukin-6
IL-10: Interleukin-10
LR: Lactated ringers
NGAL: Neutrophil gelatinase-associated lipocalin
PPV: Proportion of perfused vessels
TGA: Therapeutic Goods Administration
